# A Tablet-Based Retinal Function Test in Neovascular Age-Related Macular Degeneration Eyes and At-Risk Fellow Eye

**DOI:** 10.1167/tvst.7.2.2

**Published:** 2018-03-01

**Authors:** Chi Yun Doreen Ho, Zhichao Wu, Andrew Turpin, David J. Lawson, Chi D. Luu, Allison M. McKendrick, Robyn H. Guymer

**Affiliations:** 1 Center for Eye Research Australia, Royal Victorian Eye and Ear Hospital, East Melbourne, Australia; 2 Department of Surgery (Ophthalmology), University of Melbourne, Melbourne, Australia; 3 Department of Computing and Information Systems, University of Melbourne, Melbourne, Australia; 4 Department of Optometry and Vision Sciences, University of Melbourne, Melbourne, Australia

**Keywords:** age-related macular degeneration, tablet-device, home monitoring

## Abstract

**Purpose:**

To determine the feasibility of a tablet-based application to detect changes in retinal sensitivity and correlations with underlying pathology in neovascular age-related macular degeneration (nAMD) eyes undergoing treatment and in at-risk fellow eyes.

**Method:**

Participants with nAMD in at least one eye were recruited, examined, and imaged using spectral-domain optical coherence tomography (SD-OCT). Retinal sensitivity was measured within the central 5° at 12 locations using a customized test delivered on an iPad. Test points were superimposed on SD-OCT locations to investigate structure/function relationships.

**Results:**

Included in the study were 53 nAMD eyes and 21 at-risk fellow eyes. In nAMD eyes, the mean retinal sensitivity was 24.1 ± 1.8 dB with reduced retinal sensitivity associated with the presence of atrophy (*P* < 0.01), retinal pigment epithelium (RPE) disruption (*P* < 0.01), and absent ellipsoid zone (EZ) (*P* < 0.01), but not with the presence of subretinal fluid (*P* = 0.94) nor intraretinal fluid (*P* = 0.52). In at-risk eyes, the average retinal sensitivity was 28.8 ± 0.6 dB, with reduced sensitivity significantly associated with the presence of drusen, atrophy, RPE disruption, and absent EZ (*P* < 0.01).

**Conclusion:**

The tablet-based test of retinal sensitivity was able to be performed by an elderly cohort with nAMD. The ability to correlate differences in sensitivity with pathology is encouraging when considering using the tablet devices as a home monitoring tool with remote surveillance. Dual pathology often present with retinal fluid confounded our ability to correlate fluid with sensitivity.

**Translational Relevance:**

These findings highlight the potential of tablet-based devices in performing visual function measures as a home monitoring tool with remote surveillance for the earlier detection of nAMD.

## Introduction

The introduction of anti-vascular endothelial growth factor (anti-VEGF) has revolutionized the management of neovascular age-related macular degeneration (nAMD), with a reduction of more than half the annual incidence rate of blindness from this late complication.^1^ It is well established that one of the major factors influencing final visual outcome in individuals with nAMD is visual acuity at time of presentation and treatment.^2–6^ Despite better community awareness of the disease, it is common for vision loss in the first eye to go unnoticed when the fellow eye has good vision. This results in delays in presentation and treatment.^7,8^ Therefore, the ability to detect reduced visual function at its earliest manifestation is imperative for better outcomes given the success of anti-VEGF treatment.

In eyes that are already undergoing treatment, assessment of disease activity is achieved by measuring visual acuity and assessing fluid status on spectral-domain optical coherence (SD-OCT) scans during clinic visits. It would be advantageous if patients could monitor their own disease activity at home with access to remote surveillance to determine their need for review and treatment. The current standard of care for eyes at risk of developing nAMD is the use of the Amsler grid, where a new distortion should alert individuals to seek medical attention.^7^ Evidence has shown that the Amsler grid is limited in its detection and diagnostic accuracy.^9^ Factors that contribute to its poor performance include high false-negative rates, underdetection of scotomas 6° or less in diameter, noncompliance in self-monitoring, or of more concern, the failure to report even when changes are noted due to the lack of confidence in self-monitored symptoms.^7,9–12^ These issues highlight the need for a different approach in both test design and monitoring strategies to achieve early detection of nAMD or progression of disease activity. To date, some advances have been made using novel technologies, such as the ForeseeHome device^13^ and the MyVisionTrack application,^14^ which are based on principles of hyperacuity and can be performed in the home and monitored remotely.

There is a potential for visual function tests to be used on portable devices as a home-based, self-monitoring tool with the ability to download data to a remote surveillance hub or to a clinician. The ability to detect pathology associated with neovascular disease activity as a change in retinal sensitivity has been shown using microperimetry.^15^ Advances in technology in resolution and luminance ranges on portable tablet devices such as the iPad (Apple, Inc., Cupertino, CA) now offer the opportunity to develop applications to test retinal function, similar in principle to clinic-based microperimetry, but on small, accessible, commonplace domestic tablets.^16^ Such a tool might allow early detection of neovascular complications or potentially renewed activity of nAMD so that treatment could be delivered with minimal delay.

We have developed an iPad-based application to measure retinal sensitivity using the open-source platform, PsyPad.^16^ The PsyPad platform has previously been used to develop a perimetric test of central visual function,^16^ and the performance of that test when used in participants with intermediate AMD has been previously reported by Wu et al.^17^ The aim of the present study was to determine the feasibility of using the tablet-based test, now with a larger testing area and increased test points, in a different subset of patients, that is, people receiving ongoing anti-VEGF for nAMD eyes and in high-risk fellow eyes of those being treated for nAMD who had not yet developed neovascular complications. Different from the previous study, we also explore functional correlations with underlying pathologic changes seen in nAMD eyes to determine whether there would be potential to detect differences in sensitivity between retinal areas with and without pathologic features associated with AMD. If the tablet-based tool appeared feasible to use in this population in a clinic setting, and there was a correlation with pathology, then the possibility of using it in the home for remote monitoring for both early detection of nAMD and ongoing monitoring once anti-VEGF treatment had commenced could be further explored.

## Methods

This study was approved by the Human Ethics Committee of the Royal Victorian Eye and Ear Hospital (RVEEH) and was conducted in accordance with the Declaration of Helsinki. Written informed consent was obtained from all participants following explanation of the nature of the study.

### Participants

Participants were recruited from the medical retinal clinics at RVEEH, a major public teaching hospital. Inclusion criteria required at least one eye with nAMD to be undergoing anti-VEGF treatment and to have a best-corrected visual acuity (BCVA) of 20/80 or better and a refractive error of ±5.00 diopter (D) or less. Bilateral nAMD cases had both eyes enrolled. Fellow eyes without nAMD (but with features consistent with AMD, either medium or large drusen or geographic atrophy [GA] according to Beckman classification)^18^ were included in the at-risk fellow eye study. Exclusion criteria included any other cause of choroidal neovascularization (CNV) and the presence of any other ocular pathology such as cataract, glaucoma, amblyopia, or any neurologic or systemic disease that could compromise vision. Participants were also excluded if they had any physical or mental impairment preventing them from performing the visual function test.

### Procedure

All participants who met the inclusion criteria underwent measurement of BCVA using a logMAR chart at 4 m in standard room lighting, followed by an examination on the tablet visual field–testing application (created using PsyPad), which they performed three times in a room with lights switched off before pupils were dilated. SD-OCT scans were performed after the tablet-based test, followed by clinical ophthalmic examination. Some participants repeated all the tests when they returned for their next appointment following the same clinical procedure as stated above.

### Measurement of Retinal Sensitivity Using an iPad Application

**Figure 1:**
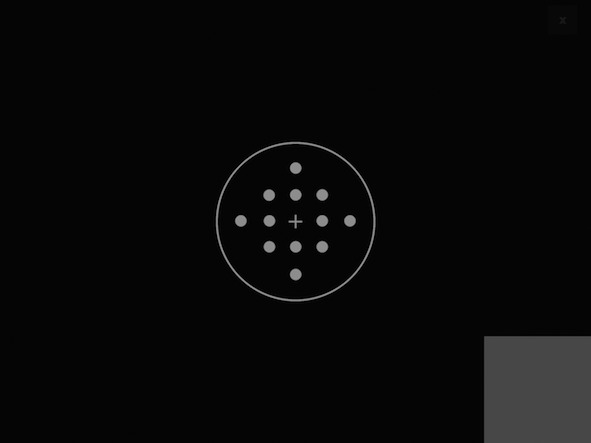
The customized test designed to test the central 5° of vision using the open-source PsyPad platform to measure retinal sensitivity. The location of the 12 test stimuli (gray) and fixation target (central cross) is shown. A gray square at the bottom right-hand corner acted as a button for participants to press when they could detect a stimulus.

A customized application (Fig. 1) that measures retinal sensitivity within the central 5° of vision was designed on an open-source platform, PsyPad, and implemented on an iPad 3 tablet (Apple, Inc.), enabling display of images at desired timing and the implementation of a staircase thresholding algorithm.^17^ The images included a uniform background (luminance of 1.27 cd/m^2^), a central cross-fixation target, and test stimuli that were circular white targets (both of which were Goldmann Size III or 0.43°) of specific luminance levels at 1-dB increments; the maximum and minimum luminance of stimuli, set at 317.50 and 1.52 cd/m^2^, respectively, provided a dynamic range of 31 dB. Stimuli were presented randomly for 200 milliseconds without any cues at 12 locations, 1° apart in horizontal and vertical meridian within the central 5° of vision. A 4-2 staircase strategy was used to obtain the threshold measurements, with the initial stimuli set at 24 dB for all tests.^17^

Participants were seated at a table in front of the tablet device and adjusted to the correct viewing distance of 50 cm by the examiner. Participants were instructed to wear their distance refractive correction with a +2.00 D near addition placed over their correction. Before formal measurement of thresholds, the examiner explained the procedure. The fellow untested eye was occluded. If a participant had both eyes tested, the right eye would be tested first followed by the left eye (regardless of whether it was the treatment eye or at-risk fellow eye). The room light was then switched off. There was no strict formal adaptation to the ambient illumination or background luminance of the screen. A gray square at the bottom-right corner was designated as an on-screen response button. All participants performed a practice examination to familiarize themselves before undertaking two formal examinations of the test eye. The data from these two tests were used for determining the intrasession test-retest repeatability. All responses were recorded in log files and sent to a specified server. Both pointwise sensitivities (PWS) and averaged sensitivity of all 12 locations (termed “mean sensitivity” in this study [MS]) were used for the analysis.

### SD-OCT Image Analysis

**Figure 2:**
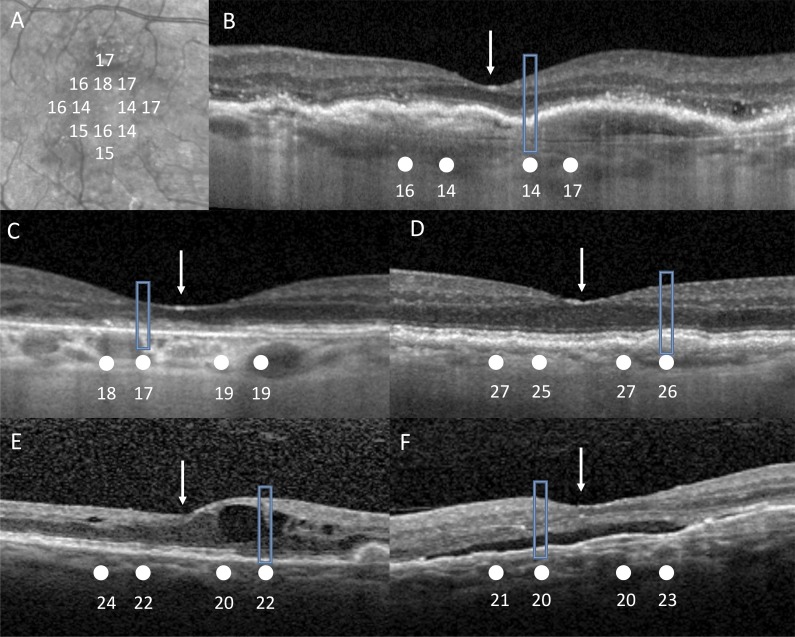
Infrared images (A) were first manually measured true to scale of test point locations (white) using measuring calipers on Heidelberg Eye Explorer. Each stimulus location and retinal sensitivity in decibels (white) was then manually transferred using measuring calipers onto the corresponding location of the SD-OCT B-scan (B) true to scale. White arrow corresponds to fovea. Rectangular boxed area shows area of pathology captured by test point. (B) Depicting area of fibrous pigment epithelial detachment (PED). (C) Depicting area of absent EZ. (D) Depicting area of drusen. (E) Depicting area of intraretinal fluid and absent EZ. (F) Depicting area of subretinal fluid and PED.

SD-OCT volume scans were obtained using an HRA+OCT device (Spectralis; Heidelberg Engineering, Heidelberg, Germany). Volume scans were performed over the central 10° × 10° area, with seven equally spaced horizontal B-scans used. The degree of arc (0.43°) that the test point subtended at 50 cm was converted to a retinal measurement based on an average-length eye of 23 mm.^19^ A software platform (Heidelberg Eye Explorer; Heidelberg Engineering) was then used to manually plot the PsyPad test points using their measuring calipers to the corresponding location on SD-OCT infrared images and a B-scan true to scale (Fig. 2). After transferring each test point location to the SD-OCT B-scan, the status of the SD-OCT structure was categorized according to the presence of retinal pathologic findings by one grader who was blinded to retinal sensitivity. Pathologic features included drusen; GA; pigment epithelial detachment (PED), both serous and fibrovascular; subretinal fluid (SRF); and intraretinal fluid (IRF). The retina was graded intact if the hyperreflective lines, RPE, ellipsoid zone (EZ), and external limiting membrane (ELM) were present on SD-OCT. Test locations were excluded if they were located at margins of retinal pathology.

### Statistical Analysis

A generalized estimation equation (GEE) was used to examine whether there were significant differences in PWS and average sensitivity between test-retest intrasession examinations in treatment eyes and at-risk eyes. A GEE model was used to include eye laterality as both eyes of some participants were included in this study. Bland-Altman plots were used to visually inspect the test-retest repeatability, and the coefficients of repeatability (CoR) and 95% limits of agreement (95% confidence interval [CI]) were determined for PWS and average sensitivity in each of the groups (treatment and at-risk) and in different BCVA groups. We used a GEE model to evaluate retinal sensitivity corresponding to retinal structure. This analysis method was used as there were often multiple pathologies in treatment eyes, and if more than one pathologic feature was found at one location, interactions could be minimized to determine the significance of a single pathology. All statistical analyses were performed using commercially available statistical software (SPSS, software version 24; IBM/SPSS, Inc., Chicago, IL).

## Results

A total of 53 subjects (74 eyes) were enrolled in the study. Fifty-three eyes from 53 subjects were treatment eyes. Twenty-one of these subjects also had their fellow at-risk eyes tested. Table 1 shows the demographics of our study cohort.

**Table 1:**
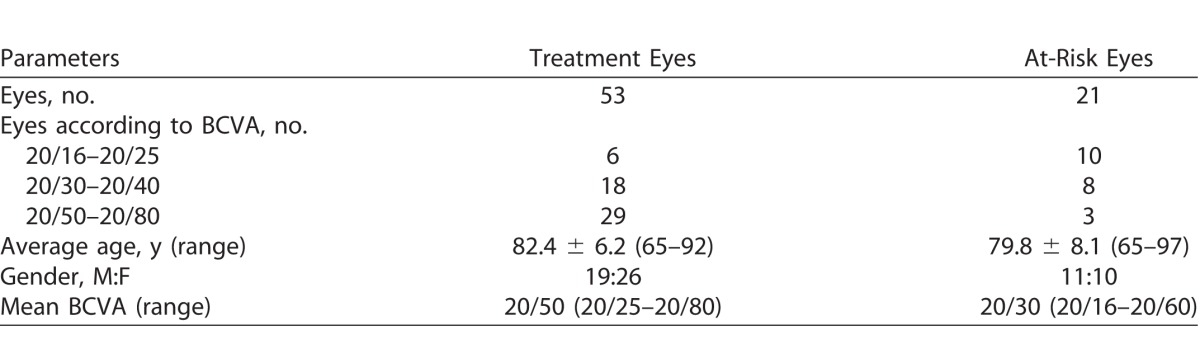
Demographic and Clinical Findings of Participants

### Intrasession Test-Retest Repeatability of Retinal Sensitivity on the iPad

Each test on the iPad took an average of 2.08 ± 0.4 minutes for the treatment eyes and 1.92 ± 0.5 minutes for the at-risk eyes. The average test duration between each of the two tests performed within the same session for at-risk eyes was not significantly different (*P* = 0.44). In treatment eyes, however, participants performed the second test more quickly (2.03 ± 0.4 minutes) compared to the first test (2.13 ± 0.4 minutes, *P* = 0.02).

**Figure 3:**
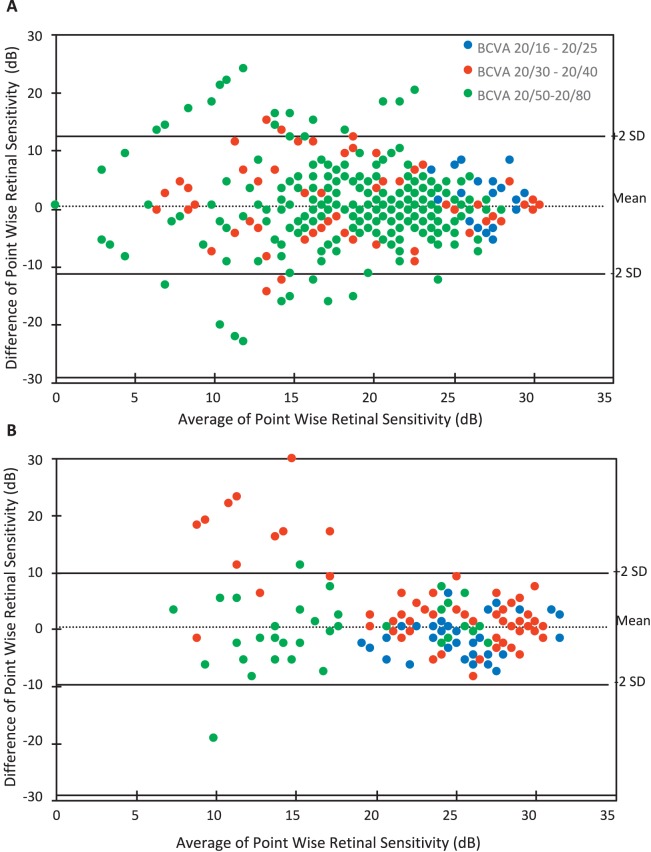
(A) Bland-Altman plot of mean PWS measurements of intrasession results on treatment eyes across all BCVA categories obtained using iPad application. (B) Bland-Altman plot of mean PWS measurements of intrasession results on at-risk eyes across all BCVA categories obtained using the iPad application.

Test-retest CoR and limits of agreement across all BCVA categories in treatment and at-risk eyes were determined by Bland-Altman and can be seen in Tables 2 and 3, respectively. Bland-Altman plots (Figs. 3A and 3B) were used to examine the agreement. The CoR of the treatment and at-risk eyes were 12.3 and 10.2 dB, respectively. Furthermore, an additional analysis was performed from one eye (the first tested eye) of each participant, and similar results of CoR were obtained (data not shown).

**Table 2:**
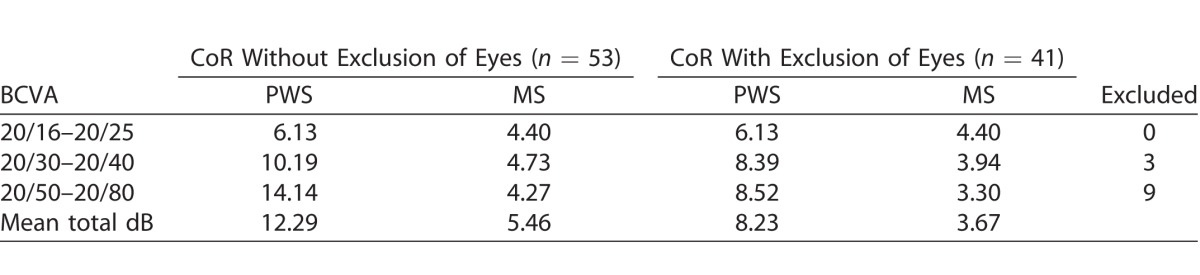
CoR for BCVA Groups in Treatment Eyes

**Table 3:**
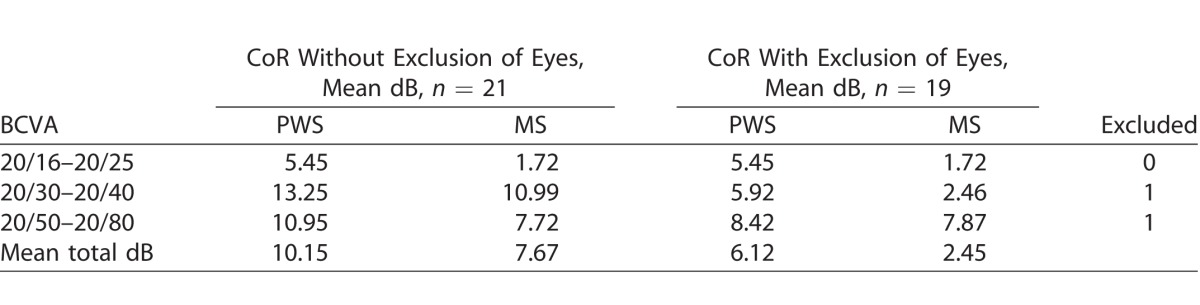
CoR for BCVA Groups in At-Risk Eyes

A subanalysis was conducted to examine intrasession repeatability when excluding those eyes that had two or more locations with ≥2 SD from mean total PWS difference between intrasession tests (*n* = 12 treatment eyes excluded [2 SD = 13 dB]; *n* = 2 at-risk eyes excluded [2 SD = 11 dB]). The CoR improved to 8.2 and 6.1 dB for treatment and at-risk eyes, respectively (Tables 2 and 3).

### Correlation of SD-OCT Structure to Retinal Sensitivity Function on the iPad

In treatment eyes, a total of 628 test locations in 53 examined eyes were evaluated according to their structural correlates on SD-OCT B-scan. Pathology was present at all locations, with no test locations over normal retina. Eight locations were not included as they fell in marginal zones between different pathologies. In at-risk eyes, a total of 244 retinal sensitivity test locations in 21 examined eyes were evaluated according to their structural correlates on SD-OCT B-scans. Eight locations were not included as they fell in marginal zones between different pathologies.

Using GEE analysis, the structure and corresponding sensitivity values for treatment eyes and at-risk and treatment eyes are represented in Tables 4 and 5, respectively.

**Table 4:**
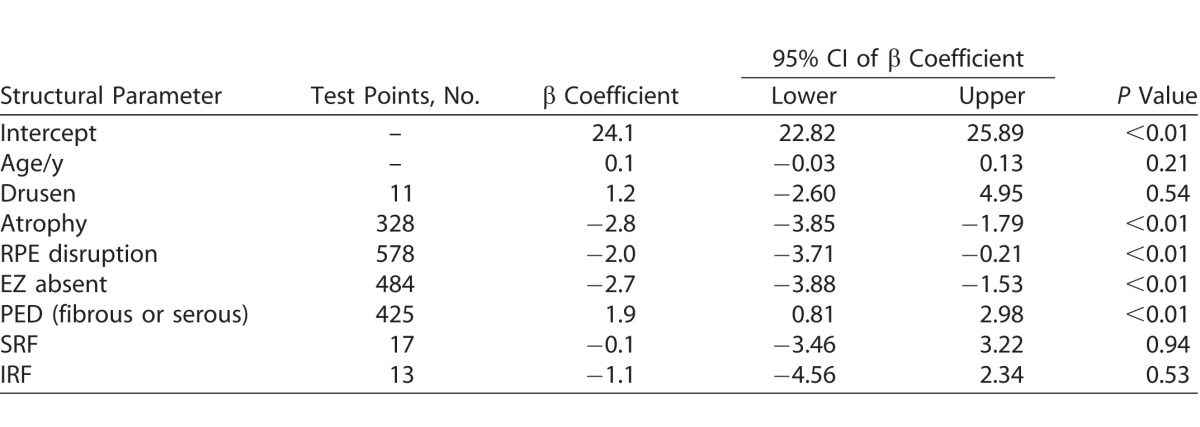
GEE Model for Retinal Sensitivity in Treated nAMD Eyes

**Table 5:**
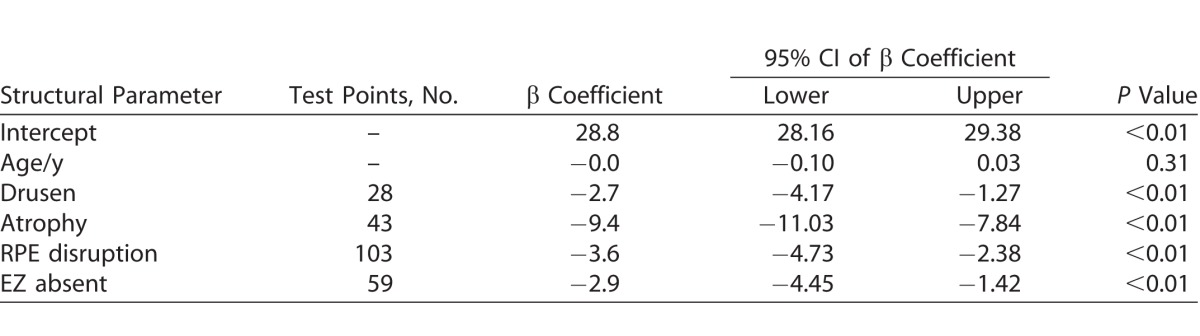
GEE Model for Retinal Sensitivity of At-Risk Eyes

The intercept coefficient of treated nAMD eyes was 24.1 dB, representing the estimated sensitivity after accounting for factors as listed in Table 4, notably lower than that of at-risk eyes (see Table 5). There was a significant association between retinal sensitivity and areas of atrophy (*P* < 0.01), RPE disruption (*P* < 0.01), and absent EZ (*P* < 0.01), but not with areas of drusen (*P* = 0.54), SRF (*P* = 0.94), or IRF (*P* = 0.53).

In the at-risk eyes, there was a significant association between retinal sensitivity and drusen (*P* < 0.01), atrophy (*P* < 0.01), RPE disruption (*P* < 0.01), and EZ absent (*P* < 0.01), but not with age (*P* = 0.31).

## Discussion

Our study aimed to evaluate the feasibility of a tablet-based application that tests macular function in a cohort of participants undergoing treatment for nAMD and their at-risk fellow eyes. In addition to determining the repeatability of such a tool, we also determined if we could detect changes in retinal sensitivity and whether sensitivity correlated with underlying pathology. We tested our tablet-based application in a clinic setting as a proof-of-principle feasibility study, but it is anticipated that such a device could be used as a tool for home monitoring.

With regard to intrasession repeatability of the test, we found a proportional relationship between poorer BCVA and the number of locations with a ≥2 SD difference in sensitivity between two functional tests for both the treatment and at-risk participants, which indicates that the test is harder to do with worse visual acuity. Furthermore, when grouped according to BCVA, we noted that there was a proportional relationship to CoR in both treatment and at-risk eyes; that is, a larger CoR was associated with poorer vision. Compared to results obtained by Macular Integrity Assessment (MAIA) and Nidek microperimeter-1 (MP-1) in participants with similar BCVA in studies conducted by Wu et al.^20^ and Chen et al.,^21^ our pilot study showed that the iPad had a poorer intrasession test-retest repeatability. Chen et al.^21^ reported PWS CoR of 5.6 dB using MP-1 with 68 test stimuli on participants with any macular disease with BCVA 20/40 or better. We previously reported PWS CoR to be 4.4 dB in intermediate AMD patients with BCVA of 20/60 or better using MAIA.^20^ The CoR obtained in this study were higher than our previous study with the tablet-based application using a similar test protocol but with fewer test locations and larger fixation target. However, only participants with intermediate AMD were included in our previous study; hence, the average BCVA in that study was better than in this study.^17^ Repeatability can be improved if we excluded individuals who were poor performers, which we defined as those that returned ≥2 points with ≥ 2 SD PWS difference between the two intrasession examinations.

We noted a better test-retest repeatability in mean sensitivities across all BCVA groups compared to PWS. Averaged values are useful in providing an overall representation of the retinal sensitivity over the area and reduces test-retest confidence limits; however, this approach would not be as sensitive at detecting localized pathologic changes.^20–24^ Pointwise analysis of the central macula will allow detection of localized changes occurring at each test stimulus site that is likely to be important for early detection of nAMD or recurrent neovascular activity.

There are several potential reasons that could contribute to the high PWS test-retest repeatability, such as learning effect, the well-studied increase in test-retest variability that is a feature of perimetry once sensitivity decreases, test distance stability, lack of false-positive and negative markers, fixation tracker, fixation target, fatigue, and sample population, because some of these factors have been noted previously to be significant factors in microperimetry repeatability.^25–27^ To minimize potential learning effects, all participants underwent a practice examination prior to the final two test-retest examinations. With this in place, we did not see a uniform improvement, nor was there a significant difference between these final two tests in the intrasession testing, suggesting that there was minimal learning effect after the first trial test. Our study population had a mean age of 80 years, a higher average age than all previous studies evaluating test-retest repeatability,^20,21^ with some individuals undergoing examinations up to six times in one setting when both eyes were eligible. Nevertheless, we found that these elderly participants were capable of performing the test and in a test time that took on average only 2 minutes. In our study, we noted that CoR had a clear proportional relationship to BCVA in both at-risk and treatment. This should be factored into protocol designs for further testing of the iPad application. Luminance and target size are important factors for fixation, especially in AMD participants, and may need to be modified on the iPad application to optimize the performance.^28^

We explored the relationship between local pathologic change and retinal sensitivity to determine the nature of the structure/function correlations. The results of our study suggest that the iPad application is able to detect differences in retinal sensitivity related to the underlying pathologic changes associated with AMD, similar to that reported with formal microperimetry.^29–32^ When looking at our cohort of treatment eyes with nAMD, we noticed a general lower mean retinal sensitivity of 24.1 dB compared to the at-risk fellow eye (28.8 dB). While sensitivity associated with areas of atrophy, RPE disturbance, and absent EZ had significant reductions in mean retinal sensitivities (Table 5), we were unable to show significant reduced sensitivity in areas with IRF and SRF compared to an averaged nAMD eye. A possible explanation for this is that in a treated nAMD eye there was more often than not dual pathology, and as such, relative sensitivity was studied rather than sensitivity of an isolated single pathology. Furthermore, there was also a relatively small sample size of test locations over IRF and SRF. However, we wish to highlight that retinal sensitivity over areas of IRF and SRF were still lower than that of at-risk fellow good eyes. Unlike results from previous studies,^15,29^ we did not detect reduced retinal sensitivity over locations of PED (either serous or fibrous), even though retinal sensitivity over these locations were still lower than that of at-risk fellow good eyes. A possible explanation is that in atrophy, RPE disruption and loss of photoreceptor integrity reflected in a missing EZ is associated with worse retinal sensitivity than PED, over which the retina can still be intact. Future work would require studying treatment eyes earlier before multiple pathologies played a role.

In at-risk eyes, we could demonstrate that in areas with no AMD pathology, a retinal sensitivity of 28.8 dB was obtained, similar to results obtained with MAIA.^17^ Sensitivity recorded in areas of drusen and atrophy in at-risk eyes showed a significant relative reduction in sensitivity as has been reported using the MAIA. We also found that areas of RPE disturbance and absent EZ in at-risk eyes returned reduced retinal sensitivity, more so than that of drusen alone.^22,30,33,34^

The findings from our study have important implications that can be considered for potential future improvements. We found that test-retest repeatability on the iPad was influenced by BCVA. Further improvements to the test design, such as larger fixation target, could improve function in those with lower BCVA. If the aim is to be able to detect local changes, then an analysis of PWS rather than averaged sensitivities will be required, albeit with inherently worse test-retest reliability. We found detectable reductions in retinal sensitivity over local retinal pathology associated with AMD.

Our study has a few limitations. First, the plotting of test points to their corresponding SD-OCT locations were of approximate measurements using an average retinal eye length of 23 mm, and the equally spaced horizontal SD-OCT B-scans may not have directly corresponded precisely to all test points. Furthermore, given that we could not monitor the state of fixation, some imprecision in the mapping between retinal sensitivity and anatomical location is possible. However, the pathology noted in our subjects fell across multiple test points and would potentially minimize errors arising from such approximations. Future studies could use vertical and horizontal line SD-OCT B-scans to allow for more precise spatial correlation. In our study, we tested both eyes in some participants. We wanted to reflect real situations in which some patients would require both eyes to be tested. We expect learning effects to influence CoR, and acknowledge this as being a reflection of real practice; therefore, there may be a difference between those testing only one eye compared to two eyes. Our study was designed as a feasibility study, with only a limited number of subjects with IRF and SRF. Further longitudinal studies will require a larger sample size to evaluate whether a decrease in sensitivity is associated with the first presentation of new pathology before there are irreversible changes. Ideally, development of new IRF or SRF would be able to be detected as a change in retinal sensitivity so that this tool could be used in a high-risk group to self-monitor at home.

Given that anti-VEGF therapy is widely available and that it is now clear that treatment outcomes are improved if initiated before irreversible damage to the retina occurs because delay limits the ability to improve presenting vision, there is an increased need for reliable self-monitoring tools to facilitate early detection of CNV and prompt referral for treatment. In addition, it may be possible to reduce the burden of long-term treatment by reducing clinician reviews to determine disease activity if this could be detected by a similar monitoring tool. With improvement in technology, there is a potential to deliver tests on portable devices that can be used at home, with surveillance offered remotely.

The results from our proof-of-concept study have shown that the iPad test of retinal sensitivity was able to be performed by elderly people with nAMD in at least one eye and in the fellow at-risk eye with a very short test time. Furthermore, we found differences in retinal sensitivity between locations of intact retina to that of retinal pathology associated with AMD. This opens up the possibility that such tests might be able to detect changes in sensitivity with changing pathology. Longitudinal studies at home, in people at risk of nAMD and those undergoing treatment, will be needed to determine the sensitivity and specificity of any association with these changes.
